# Treatment decisions for intermediate-sized brain metastases in or near the motor cortex among the neuro-oncology community

**DOI:** 10.1016/j.bas.2025.104278

**Published:** 2025-05-12

**Authors:** C.A.C. Jessurun, D. Brandsma, A. Compter, P.C. de Witt Hamer, R.J.A. Nabuurs, A. Kloet, M.L.D. Broekman, P.V. ter Wengel

**Affiliations:** a Department of Neurosurgery, Leiden University Medical Center, Albinusdreef 2, 2333ZA, Leiden, Zuid-Holland, the Netherlands; b Department of Neurosurgery, Haaglanden Medical Center, Lijnbaan 32, 2512VA, The Hague, Zuid-Holland, the Netherlands; c Department of Neuro-oncology, Netherlands Cancer Institute-Antoni van Leeuwenhoek Hospital, Amsterdam, the Netherlands; d Department of Neurosurgery, Brain Tumor Center Amsterdam, Amsterdam University Medical Center, Vrije Universiteit Amsterdam, Amsterdam, the Netherlands; e Department of Neurology, Massachusetts General Hospital, Harvard Medical School, 55 Fruit Street, 02114, Boston, MA, United States

**Keywords:** Brain metastases, Motor cortex, Surgery, Radiation therapy, Radiosurgery

## Abstract

**Purpose:**

Currently, there is lack of consensus regarding the optimal treatment strategy (surgery versus radiotherapy) for intermediate-sized (2–4 cm) brain metastases (BM), especially those located in or near the motor cortex. This survey aims to gain insight into treatment decisions for these BM among the Dutch multidisciplinary neuro-oncology community.

**Methods:**

An electronic survey was distributed among neurosurgeons, radiation oncologists, neurologists, and medical oncologists in The Netherlands. The survey comprised 13 questions regarding physician's practices and questions about treatment decisions for BM in or near the motor cortex using statements and three theoretical patient cases.

**Results:**

Tumor size (n = 34, 89 %), degree of neurological deficit (n = 31, 82 %), and the need for (temporarily) discontinuation of immunotherapy because of dexamethasone dependence (n = 30, 79 %) were highlighted as the most important factors to steer the treatment decision to radiotherapy or surgery. When divided by specialty, 15 neurologists (33 %), 14 radiation oncologists (30 %), 12 neurosurgeons (26 %), and 5 medical oncologists (11 %), some variability about the importance of factors exists. The respondents suggested a median cutoff size of 3,5 cm for conducting surgery on BM.

**Conclusion:**

Surgical resection is preferred in patients with larger tumors, with neurologic symptoms that are unresponsive to dexamethasone, and in patients receiving immunotherapy. Future investigations should compare the effectiveness of surgical resection and stereotactic radiosurgery, and the effects on survival and quality of life in patients with BM in or near the motor cortex in a prospective and preferable randomized manner.

## Highlights

• Treatment choices for BM near the motor cortex vary among specialists.
• Tumor size, neurological deficits, and immunotherapy use impact decision-making.
• A median cutoff size of 35 mm was suggested by respondents for conducting surgery.
• Neurosurgeons prefer surgery for smaller BM than radiation-oncologists (20 vs 35 mm).
• A lack of consensus highlights the need for randomized studies on treatment efficacy.

## 1 Introduction

Brain metastases (BM) are the most frequently occurring intracranial tumors, affecting approximately 10–30 % of patients with systemic malignancies. Lung cancer, breast cancer, and melanoma have the highest susceptibility to develop BM (Amsbaugh and Kim, 2023). The median survival time for BM patients ranges from 8 to 16 months depending on the type of tumor, number of brain metastases, presence of systemic metastases, and age and clinical condition of the patient (Sperduto et al., 2020).

In recent years, the survival rates for BM patients of the abovementioned primary tumor types have improved, partly due to the introduction of new treatment modalities including targeted therapy and immunotherapy with Immune Checkpoint Inhibitors (ICIs) (Di Giacomo et al., 2023). Treatment with ICIs shows response rates ranging from 15 to 30 % in most solid tumors to 45–60 % in melanoma patients (Das and Johnson, 2019; Zhang et al., 2021). Currently, more than 40 % of cancer patients are eligible for ICI treatment with one of the seven approved agents in the United States (Haslam and Prasad, 2019). Initial clinical trials for immunotherapy agents in metastatic cancer patients excluded BM patients, because of the classic but outdated presumption that the brain is an immune-privileged environment. Most cancer patients develop BM later during their clinical course, however 60–72 % of lung cancer patients, 2–4 % of breast cancer patients and 3–10 % of melanoma patients present with BM at their initial systemic cancer diagnosis (Füreder et al., 2018; Agazzi et al., 2004; Salvati et al., 1995; Giordana et al., 2000; Berghoff et al., 2016). With the new treatment paradigms, these patients will more often receive ICIs for systemic cancer next to local therapy for their BM, which is also reflected in the inclusion of more BM patients in clinical trials nowadays (Cagney et al., 2017; Brahm et al., 2020; Mansfield et al., 2020; Paz-Ares et al., 2019; Rudin et al., 2020; Kluger et al., 2019; Tawbi et al., 2018).

In case of oligometastatic BM, local treatment options for BM include stereotactic radiosurgery, stereotactic radiotherapy, and surgical resection (StatPearls, 2023, 2023^2023^). For more than fifty years, the steroid dexamethasone (DXM) has been consistently used in patients with brain tumors to relieve symptoms associated with peritumoral edema and increased intracranial pressure. It has also been employed both in a prophylactic manner perioperatively and during radiation therapy (Galicich et al., 1961; Jessurun et al., 2019). Stereotactic radiosurgery (SRS), with or without upfront surgical resection, is considered the standard treatment of care for solitary or oligo-metastases. Stereotactic radiosurgery is preferred for smaller metastases (<2 cm diameter), while surgical resection followed by adjuvant radiotherapy is preferred for larger metastases (>3 cm diameter). However, there is currently a lack of consensus regarding the optimal treatment strategy for the intermediate-sized BM, roughly 2–4 cm in maximal diameter (Krist et al., 2022).

When determining the optimal treatment strategy, it is important to consider the risks and benefits associated with each treatment, as well as their impact on steroid use and the potential interference with immunotherapy. Especially for patients with tumors in eloquent regions including the central motor cortex, the preservation of quality of life in the often short remaining lifespan of BM patients should be considered, next to the therapeutic efficacy. Even small BM in or near the motor cortex can be symptomatic, and the risk of neurologic complications after both surgery and/or radiotherapy might be more ominous than in other non-eloquent areas of the brain (Weil and Lonser, 2005). Surgery is a more invasive approach, yet it yields more immediate relief from symptoms compared to the frequently transient exacerbation of neurological issues caused by increased edema following radiotherapy.

This survey aims to gain insight in the clinical equipoise regarding differences in treatment decisions for BM in or near the motor cortex among the Dutch multidisciplinary neuro-oncology community.

## 2 Methods

### 2.1 Study design and survey

An electronic survey using the online tool SurveyMonkey (SurveyMonkey, San Mateo, California, USA; http://www.surveymonkey.com) was distributed among neurosurgeons, radiation oncologists, neurologists, and medical oncologists nationally (Supplementary Material 1).

The survey comprised 13 questions divided into three parts regarding physician's practices and questions about treatment decisions for BM in or near the motor cortex using statements and three theoretical patient cases. The first part consisted of four general questions about the physician's demographics. The second part consisted of a table with ten statements about the preferred treatment options for BM in the motor cortex based on factors that one might consider of importance in deciding on radiotherapy or surgery, and a ranking of the three most important factors contributing to the treatment decisions. Radiotherapy will cover stereotactic radiosurgery and stereotactic radiotherapy in this survey. These factors were predefined by the authors of this study based on clinical experience and national BM guidelines (Richtlijnendatabase and Hersenmetastasen, 2017). Furthermore, the participants of the survey were requested to identify the tumor's cut-off size at which they would opt for surgery as the preferred treatment. For the third part of the survey, the respondents were presented with three theoretical patient cases with tumors in or near the motor cortex to gain more insight into the statements about decision-making regarding BM treatment. In addition, this survey provided us with more insight into possible different treatment preferences between radiation oncologists, neurosurgeons, neurologists, and oncologists.

The respondents were informed in advance that this was an anonymous web-based survey and gave informed consent for using the data collected by the survey. Ethical approval was not sought for the present study, as distributing a one-time survey to a healthcare provider group is not subject to the Medical Research Involving Human Subjects Act in the country where the study was executed (The Netherlands).

### 2.2 Case descriptions

#### 2.2.1 Case 1

‘A 50-year-old male with a known history of non-small lung cancer (NSCLC) presented with weakness of the right arm for three weeks. MRI of the brain showed a 2 cm metastasis in the left motor cortex. Further imaging revealed a persistent small stable mass in the lung and no systemic metastases.’

#### 2.2.2 Case 2

‘A 50-year-old male with no oncological history presented with weakness of the right arm for three weeks. MRI of the brain showed a 4 cm metastasis in the left motor cortex. Further imaging showed a mass in the lung and no systemic metastases. A biopsy of the lung mass revealed NSCLC.’

#### 2.2.3 Case 3

‘A 50-year-old male without oncological history presented with weakness of the left arm for three weeks. MRI of the brain showed a 3 cm metastasis in the right motor cortex. Further imaging showed a mass in the lung and no systemic metastases. A biopsy of the lung mass revealed NSCLC.’

### 2.3 Data collection and analysis

The survey was open from 22 December 2022, through March 22, 2023. The survey was distributed by the electronic mailing lists of the Dutch Neuro-Oncology Society (LWNO) in The Netherlands (n = 255). LWNO is a multidisciplinary, national network of neurologists, neurosurgeons, medical and radiation oncologists, neuroradiologists, neuropathologists, neuropsychologists, and specialized nurses involved in care for neuro-oncological patients. Collected data was assessed qualitatively. Basic demographics were summarized using counts and percentages for nominal variables and means, medians, and ranges for continuous variables.

## 3 Results

### 3.1 Respondent characteristics

The survey was filled out by 53 respondents of which six respondents were excluded as they were not the intended target group (physicians involved in treatment decision-making) of this survey, resulting in a total of 47 respondents. The completion rate of this survey was 70 % (n = 33). The response rate of the survey was 20 % (52 out of 255 LWNO members who received the survey invitation).

The majority of the respondents worked in an academic hospital (n = 23, 49 %) or subacademic/teaching hospital (n = 17, 36 %; Table 1). The median years of experience as a medical specialist was 10.5 years.

**Table 1:** Results of physician information retrieved by survey.

Question	Number of respondents (%)	Answers	Number (%)
Institution	47 (100)	Academic hospital	23 (49)
		Subacademic/teaching hospital	17 (36)
		Community hospital	7 (15)
		Private practice	0 (0)
Years of experience as medical specialist	47 (100)	Median 10.5 (range 1–30)	–
Is neuro-oncology your sub-specialty?	47 (100)	Yes	46 (98)
		No	1 (2)
I am a:	46 (97.9)	Neurologist	15 (33)
		Radiation-oncologist	14 (30)
		Neurosurgeon	12 (26)
		Medical oncologist	5 (11)

### 3.2 Important factors in deciding on radiotherapy versus surgery

**Fig. 1:**
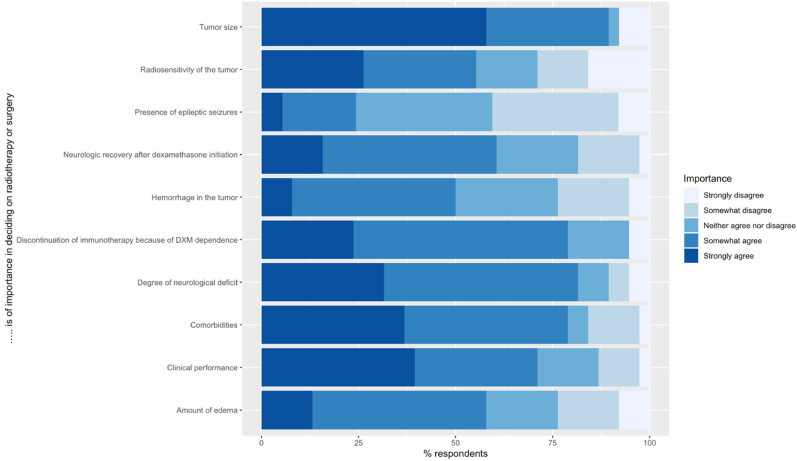
Statements about the importance of factors deciding on radiotherapy or surgery in patients with BM located in or near the mot**or cortex.** The respondents were asked to rate the importance of several factors on the treatment decision (radiotherapy or surgery) from strongly agree to strongly disagree.

**Fig. 2:**
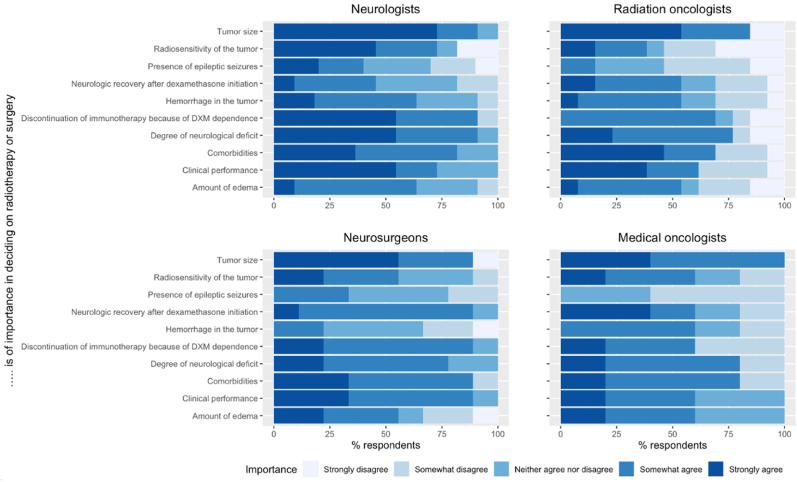
Statements about the importance of factors deciding on radiotherapy or surgery in patients with BM located in or near the motor cortex divided by the different specialties included in th**is survey.** The respondents were asked to rate the importance of several factors on the treatment decision (radiotherapy or surgery) from strongly agree to strongly disagree.

Tumor size (n = 34, 89 %), degree of neurological deficit (n = 31, 82 %), (temporarily) discontinuation of immunotherapy because of dexamethasone dependence (n = 30, 79 %), and the presence of comorbidities (n = 30, 79 %) were most often rated as important in deciding on radiotherapy or surgery (Fig. 1). More than 50 % of the neurologists strongly agreed that clinical performance is an important factor in deciding on radiotherapy or surgery. Radiosensitivity was the least often reported to be important by radiation oncologists (Fig. 2). More than 80 % of the neurosurgeons rated neurologic recovery after dexamethasone as important (strongly to somewhat) compared to about 60 % or less by the other specialties. The presence of epileptic seizures (n = 9) was the least often rated as an important factor in deciding on radiotherapy or surgery by all different specialties (Fig. 1, Fig. 2).

The top three factors ranked to mostly contribute to treatment decisions were: tumor size (n = 33, 87 %), clinical performance (n = 17, 45 %), and the degree of neurologic deficit (n = 14, 37 %; Table 2). When divided by specialty some variability in this ranking exists. Tumor size was ranked in the top three by all specialties, but neurologic recovery after dexamethasone use and edema size was ranked in the top three by medical oncologists, the need to discontinue immunotherapy because of dexamethasone dependence by neurologists, and comorbidities by radiation-oncologists.

**Table 2:** **Ranking of important factors in deciding on radiotherapy versus surgery for all respondents and divided by the different specialties included in this survey.** The top three factors are shaded grey.

Factor	All respondents	Medical oncologists (n = 5)	Neurologists (n = 11)	Neurosurgeons (n = 9)	Radiation oncologists (n = 13)
Tumor size	33 (87)	4 (80)	9 (82)	8 (89)	12 (92)
Clinical performance	17 (45)	1 (20)	5 (45)	5 (56)	6 (46)
The degree of neurologic deficit	14 (37)	2 (40)	2 (18)	6 (67)	4 (31)
Radiosensitivity of the tumor	10 (26)	3 (60)	5 (45)	1 (11)	1 (8)
Neurologic recovery after dexamethasone use	10 (26)	2 (40)	1 (9)	4 (44)	3 (23)
The need to (temporarily) discontinue immune therapy because of dexamethasone dependence	10 (26)	1 (20)	6 (55)	2 (22)	1 (8)
Comorbidities	10 (26)	1 (20)	0 (0)	4 (44)	5 (38)
Edema size	7 (18)	2 (40)	0 (0)	2 (22)	3 (23)
Hemorrhage in the tumor	3 (8)	1 (20)	0 (0)	0 (0)	2 (15)
The presence of epileptic seizures	1 (3)	0 (0)	1 (9)	0 (0)	0 (0)

The median cutoff size the respondents proposed to perform surgery for BM located in or near the motor cortex was 35 mm (interquartile range: 25–40 mm). When divided by specialty, the median proposed cutoff size was 20 mm for the neurosurgeons, 35 mm for the radiation-oncologists, 34 mm for the neurologists, and 40 mm for the medical oncologists.

### 3.3 Cases with intermediate-sized BM in or near the motor cortex and treatment decisions

#### 3.3.1 Case 1: A 2-cm-sized tumor with profound peritumoral edema

Most of the respondents chose radiotherapy as the preferred treatment (n = 25, 74 %), whereas seven respondents (21 %) chose surgery (combined with radiotherapy) as the preferred treatment.

If in this case the tumor size would be larger, present with an increase in neurologic symptoms not responsive to dexamethasone, or immunotherapy would have to be discontinued because of dexamethasone dependence, more than half of the respondents would prefer surgery over radiotherapy (n = 26, 76 %; n = 22, 65; n = 21, 62 %, respectively; Table S1) Some discrepancies existed between the different specialties and the preferred treatment switch from radiotherapy to surgery. Neurosurgeons more often maintained their preference for radiotherapy if the patient-case would have a larger tumor size (n = 2, 50 %) and were neutral to the preferred treatment option in case of neurologic worsening not responsive to dexamethasone (n = 2, 50 %). The radiation oncologists were most often neutral in their treatment preference in case of the need to discontinue immunotherapy because of dexamethasone dependence (n = 5, 57 %) although one-third of the radiation oncologists switched to surgery as the preferred treatment option (Table S2).

On the other hand, more than half of the respondents would prefer radiotherapy over surgery if the tumor would be radiosensitive (n = 22, 65 %) or if the case would show neurologic recovery after dexamethasone initiation (n = 17, 50 %; Table S1).

#### 3.3.2 Case 2: a 4-cm-sized tumor with little peritumoral edema

Most of the respondents chose surgery (combined with radiotherapy) as the preferred treatment (n = 24, 71 %), whereas ten respondents (29 %) chose radiotherapy as the preferred treatment (Table S3 and S4). Most respondents who chose radiotherapy were oncologists (n = 3, 60 %) followed by neurosurgeons (n = 3, 43 %) and radiation oncologists (n = 3, 27 %; Table S4).

Surgery was preferred by more than half of the respondents if the patient would have a larger tumor size (n = 30, 88 %), would show neurologic worsening not responsive to dexamethasone (n = 19, 56 %), or when immunotherapy would be discontinued because of dexamethasone dependence (n = 20, 59 %; Table S3). In comparison with the other specialties, two-thirds of the neurosurgeons stuck to the preferred radiotherapy if the case would have a larger tumor size, and none of the radiation oncologists switched from radiotherapy to surgery if there was a need for discontinuation of immunotherapy because of dexamethasone dependence (Table S4)**.** In case of a radiosensitive tumor, most respondents preferred radiotherapy (Table S3 and S4).

#### 3.3.3 Case 3: A 3-cm-sized tumor with medium peritumoral edema

The majority of the respondents chose radiotherapy as the preferred treatment (n = 21, 64 %), whereas eleven respondents (33 %) chose surgery as the preferred treatment. More than half of the respondents would prefer surgery if the tumor, in this case, would be larger (n = 24, 73 %), if neurologic worsening was not responsive to dexamethasone (n = 21, 64 %), and when immunotherapy would have to be discontinued because of dexamethasone dependence (n = 22, 67 %; Table S5). In comparison with the other specialties, three out of four neurosurgeons stuck to the preferred radiotherapy if the case had a larger tumor size (Table S6). Radiotherapy was preferred by nineteen respondents (58 %) if the tumor was radiosensitive, and by sixteen respondents (48 %) if the case would show neurologic recovery after dexamethasone initiation (Table S6).

## 4 Discussion

The present survey demonstrated variation in the preferences for radiotherapy or surgical resection as treatment for BM in the motor cortex, and the factors influencing treatment decisions. The tumor size, degree of neurological deficit, and the need for (temporarily) discontinuation of immunotherapy because of dexamethasone dependence were most often rated as important in deciding on radiotherapy or surgical resection. The respondents suggested a median cutoff size of 35 mm for conducting surgery on BM situated in or close to the motor cortex. The amount of edema was less often reported to be important in the treatment decision according to the survey respondents.

One important benefit of surgery is immediate mass and edema reduction, which is especially important in larger tumors (Ene and Ferguson, 2022). This reduces the duration of steroid use in patients, which potentially limits the development of steroid-induced complications and may prevent discontinuation of immunotherapy. Concurrent use of ICIs and dexamethasone leads to a decrease in overall survival in BM patients (Jessurun et al., 2021). However, resection of BM in or near the motor cortex poses a risk for increased or new neurological deficits that might significantly decrease the performance score of the patient and negatively impact the quality of life. This may limit the possibilities of receiving adjuvant therapy and/or clinical trial enrollment (Ene and Ferguson, 2022).

According to the Dutch BM guidelines (Richtlijnendatabase and Hersenmetastasen, 2017), the choice of surgical resection versus radiotherapy should be individualized based on the following factors: clinical performance of the patient, neurological symptoms, extracranial disease activity, number of BM, tumor volume, location of the tumor, surgical attainability of the tumor, need for histological/molecular diagnostics, and the patients’ wish. In general, for solitary BM, surgical resection is preferred over stereotactic radiosurgery in symptomatic lesions of >2.5 cm in diameter and tumors needing a tissue diagnosis. In most other cases, stereotactic radiosurgery is the treatment of choice due to its non-invasive character in the palliative setting (Richtlijnendatabase and Hersenmetastasen, 2017; Fuentes et al., 2018). According to the ASCO-SNO-ASTRO Guidelines, based on randomized trials including mainly BM of less than 3 or 4 cm in diameter, stereotactic radiosurgery alone should be offered to patients with one to four unresected BM, excluding (chemo-responsive) small-cell carcinoma (Vogelbaum et al., 2022).

A recent meta-analysis observed no survival difference in patients with 1–4 BM less than 4 cm between surgery with or without SRS or WBRT versus SRS or WBRT alone, but patients that underwent a resection showed a higher risk for local tumor recurrence compared to SRS alone (Krist et al., 2022). The local control of BM after stereotactic decreases with larger tumor volumes(Hughes et al., 2016; Garsa et al., 2014), similar to local control after surgery (Mahajan et al., 2017).

The median cutoff size that the respondents proposed in this survey to perform surgery for BM in or near the motor cortex was 35 mm. When divided by the different specialties included in the survey, the neurosurgeons proposed a smaller median cutoff size of 20 mm versus the proposed 35 mm of the radiation oncologists. This suggests that radiation oncologists are more likely to propose radiotherapy for larger BM than neurosurgeons.

Irrespective of the presented case in this survey, more than half of the respondents preferred surgery over radiotherapy if the case-patients would have a larger tumor, experienced neurologic worsening not responsive to dexamethasone, or would need to discontinue immunotherapy because of dexamethasone dependence. This highlights the important role of dexamethasone use in treatment decisions in BM patients.

The limitations of this national survey study should be mentioned. First of all, the survey was distributed by the LWNO which resulted in self-selected participation, which is a common limitation of survey studies. Most respondents were specialists from academic and teaching hospitals, who are actively involved in multidisciplinary decision-making regarding BM treatment. This may have led to a selection bias and a relatively low response rate of 20 %, as LWNO members from peripheral hospitals may have felt less confident in completing the survey, despite also being involved in patient care. Secondly, as the survey was executed in the Netherlands, it reflects the prevailing perspective on the treatment of BM in the Netherlands. Therefore, caution should be exercised when attempting to extrapolate these findings to other countries. In addition, due to the small sample size and response rate of 20 % no statistical analysis was possible to compare the results between the different specialties included in this survey. Nevertheless, a strength of this survey is that this is the first survey in the Dutch neuro-oncological community focused on this topic.

Until now, no definitive evidence has been shown regarding the efficacy and safety of surgical resection or radiosurgery in patients with single intermediate-sized BM in or near the motor cortex on quality of life, adverse events, and survival (Fuentes et al., 2018). Randomized studies comparing surgical resection and stereotactic radiosurgery are scarce and lack the power to draw any conclusions (Fiore et al., 2023). Thus, there is a need for future investigation in a prospective and preferably in a randomized manner into the effectiveness of surgical resection versus stereotactic radiosurgery on survival and quality of life in patients with intermediate-sized BM in or near the motor cortex. A randomized trial might be difficult as only few patients are eligible to receive both surgical resection or stereotactic radiosurgery by randomization, and in palliative setting patients often have their preferences for therapy (Fiore et al., 2023). Moreover, decision tools to support decisions between these two treatment modalities among the neuro-oncology community would be helpful.

## 5 Conclusions

This survey among the Dutch neuro-oncology community indicates the factors influencing treatment decisions for BM in or near the motor cortex: surgical resection is preferred in patients with larger tumors, with an increase of neurologic symptoms that are unresponsive to dexamethasone, and in patients receiving immunotherapy. Future investigations should compare the effectiveness of surgical resection and stereotactic radiosurgery and the effects on survival and quality of life in patients with BM in or near the motor cortex.

## Author contributions

All authors contributed to the study conception and design. Material preparation, data collection, and analysis were performed by C.A.C. Jessurun. The first draft of the manuscript was written by C.A.C. Jessurun and all authors commented on previous versions of the manuscript. All authors read and approved the final manuscript.

## Data availability

The datasets generated during and/or analysed during the current study are available from the corresponding author on reasonable request.

## Ethics approval

Ethical approval was not sought for the present study, as distributing a one-time survey to a healthcare provider group is not subject to the Medical Research Involving Human Subjects Act in the country where the study was executed (The Netherlands).

## Declaration of generative AI and AI-assisted technologies in the writing process

During the preparation of this work the author(s) used chat-gpt in order to improve the English language and readability. After using this tool/service, the authors reviewed and edited the content as needed and take full responsibility for the content of the publication.

## Funding

The authors declare that no funds, grants, or other support were received during the preparation of this manuscript.

## Competing interests

The authors have no relevant financial or non-financial interests to disclose.
