# Supplementary material for: Treatment decisions for intermediate-sized brain metastases in or near the motor cortex among the neuro-oncology community
Source: Brain Spine. 2025 May 12;5:104278. doi: 10.1016/j.bas.2025.104278 (PMC12145800; doi:10.1016/j.bas.2025.104278)
Supplement: Multimedia component 1 [file mmc1.docx]

**Supplementary material 1: survey**

**Accompanying email**

Dear sir/madam,

Because you are practicing in the field of neuro-oncology, I am inviting you to participate in this research study by completing an online survey. This survey aims to increase insight in the treatment decisions for centrally located brain metastases among the neuro-oncology community.

Your participation in the survey is completely voluntary and all of your responses will be kept confidential. No personally identifiable information will be associated with your responses to any reports of these data. The survey will take about 5-10 minutes to complete.

Thank you very much in advance for your time.

Yours sincerely,

**Survey**

Part 1 Physician Information

1. Institution:
2. University hospital
3. Subacademic/Teaching hospital
4. Community hospital
5. Private practice
6. Years of experience as medical specialist: open answer
7. Is neuro-oncology your sub-specialty: yes – no

I am a Neurosurgeon

Radiation therapist

Neurologist

Medical oncologist

Resident

Other, ……

Do you discuss each patient with brain metastases in a tumor board?

Yes

No, but >90%

No, but approximately 50-90%

No, <50%

Which specialties attend the tumor board meeting?

- Neurosurgery
- Radiotherapy
- Radiology
- Neurology
- Oncology
- Pathology
- Other, ……

**Part 2 Statements**

Do you agree or disagree with the following statements (in table):

1. The tumor size is of importance in deciding on radiotherapy versus surgery:

- Strongly agree
- Somewhat agree
- Neither agree nor disagree
- Somewhat disagree
- Strongly disagree

What would your cutoff size be to perform surgery:

1. The size of edema is of importance in deciding on radiotherapy versus surgery:

- Strongly agree
- Somewhat agree
- Neither agree nor disagree
- Somewhat disagree
- Strongly disagree

1. Haemorrhage in the tumor is of importance in deciding on radiotherapy versus surgery

- Strongly agree
- Somewhat agree
- Neither agree nor disagree
- Somewhat disagree
- Strongly disagree

1. The degree of neurologic deficit is of importance in deciding on radiotherapy versus surgery:

- Strongly agree
- Somewhat agree
- Neither agree nor disagree
- Somewhat disagree
- Strongly disagree

1. The presence of epileptic seizures is of importance in deciding on radiotherapy versus surgery:

- Strongly agree
- Somewhat agree
- Neither agree nor disagree
- Somewhat disagree
- Strongly disagree

1. Is radiosensitivity of the tumor of importance in deciding on radiotherapy versus surgery

- Strongly agree
- Somewhat agree
- Neither agree nor disagree
- Somewhat disagree
- Strongly disagree

1. Neurologic recovery after dexamethasone initiation is of importance in deciding on radiotherapy versus surgery

- Strongly agree
- Somewhat agree
- Neither agree nor disagree
- Somewhat disagree
- Strongly disagree

9. (Temporarily) discontinuation of immune therapy because of dexamethasone dependence is of importance in deciding on radiotherapy versus surgery

- Strongly agree
- Somewhat agree
- Neither agree nor disagree
- Somewhat disagree
- Strongly disagree

*Which factors contribute the most to your decision to recommend radiotherapy or surgery? (Choose a maximum of three factors)*

- *Tumor size*
- *Edema size*
- *Hemorrhage in the tumor*
- *The degree of neurologic deficit*
- *The presence of epileptic seizures*
- *Radiosensitivity of the tumor*
- *Neurologic recovery after dexamethasone use*
- *The need to (temporarily) discontinue immune therapy because of dexamethasone dependence*

**Part 3 Cases**

We would like to gain more insight in the aforementioned statements about decision making regarding brain metastases treatment by introducing three short patient cases:

**Case 1: tumor of 2cm with a lot of edema**

A 50-year-old male with a known history of non-small lung cancer presented with weakness of the right arm since three weeks. MRI of the brain showed a metastasis in the left motor cortex. Further imaging revealed a persistent small stable mass in the lung and no systemic metastases.

What would your preferred treatment be based on this case?

- Radiotherapy
- Surgery (+ radiotherapy)

Would your treatment change in case of:

|  | RT ---------------------------------------------------------------------------- Surgery | | | | |
| --- | --- | --- | --- | --- | --- |
| In case of: | Most preferred | Preferred | Neutral | Preferred | Most preferred |
| Less edema |  |  |  |  |  |
| Bigger tumor |  |  |  |  |  |
| Hemorrhage in the tumor |  |  |  |  |  |
| Neurologic worsening |  |  |  |  |  |
| Epileptic seizures |  |  |  |  |  |
| Radiosensitive tumor |  |  |  |  |  |
| Neurologic recovery after dexamethasone |  |  |  |  |  |
| Needing to discontinue immunotherapy due to dexamethasone dependence |  |  |  |  |  |

**Case 2: tumor of 4cm with little edema**

A 50-year-old male with no oncological history presented with weakness of the right arm since three weeks. MRI of the brain showed a metastasis in the left motor cortex. Further imaging showed a mass in the lung and no systemic metastases. A biopsy of the lung mass revealed non-small cell lung cancer.

What would your preferred treatment be?

- Radiotherapy
- Surgery (+ radiotherapy)

Would your treatment change in case of:

|  | RT ---------------------------------------------------------------------------- Surgery | | | | |
| --- | --- | --- | --- | --- | --- |
| In case of: | Most preferred | Preferred | Neutral | Preferred | Most preferred |
| More edema |  |  |  |  |  |
| Bigger tumor |  |  |  |  |  |
| Hemorrhage in the tumor |  |  |  |  |  |
| Neurologic worsening |  |  |  |  |  |
| Epileptic seizures |  |  |  |  |  |
| Radiosensitive tumor |  |  |  |  |  |
| Neurologic recovery after dexamethasone |  |  |  |  |  |
| Needing to discontinue immunotherapy due to dexamethasone dependence |  |  |  |  |  |

**Case 3: tumor of 3cm with medium edema**

A 50-year-old male with no oncological history presented with weakness of the left arm since three weeks. MRI of the brain showed a metastasis in the right motor cortex. Further imaging showed a mass in the lung and no systemic metastases. A biopsy of the lung mass revealed non-small cell lung cancer.

What would your preferred treatment be?

- Radiotherapy
- Surgery (+ radiotherapy)
- Would your treatment change in case of:

|  | RT ---------------------------------------------------------------------------- Surgery | | | | |
| --- | --- | --- | --- | --- | --- |
| In case of: | Most preferred | Preferred | Neutral | Preferred | Most preferred |
| More edema |  |  |  |  |  |
| Bigger tumor |  |  |  |  |  |
| Hemorrhage in the tumor |  |  |  |  |  |
| Neurologic worsening |  |  |  |  |  |
| Epileptic seizures |  |  |  |  |  |
| Radiosensitive tumor |  |  |  |  |  |
| Neurologic recovery after dexamethasone |  |  |  |  |  |
| Needing to discontinue immunotherapy due to dexamethasone dependence |  |  |  |  |  |

| Table S1 Statements about factors influencing treatment decisions in case 1 | | | | | | |
| --- | --- | --- | --- | --- | --- | --- |
| Would your treatment change in case of: | Radiotherapy most preferred | Radiotherapy preferred | Neutral | Surgery preferred | Surgery most preferred | Total number of respondents |
| Less edema | 9 (28.13) | 6 (18.75) | 15 (46.88) | 2 (6.25) | 0 (0.00) | 32 |
| Larger tumor size | 2 (5.88) | 1 (2.94) | 5 (14.71) | 20 (58.82) | 6 (17.65) | 34 |
| Intratumoral hemorrhage | 1 (3.03) | 2 (6.06) | 20 (60.61) | 10 (30.30) | 0 (0.00) | 33 |
| Neurologic worsening (not responsive to dexamethasone) | 1 (2.94) | 5 (14.71) | 6 (17.65) | 17 (50.00) | 5 (14.71) | 34 |
| Presence of epileptic seizures | 2 (5.88) | 0 (0.00) | 23 (67.65) | 9 (26.47) | 0 (0.00) | 34 |
| Radiosensitive tumor | 10 (29.41) | 12 (35.29) | 9 (26.47) | 3 (8.82) | 0 (0.00) | 34 |
| Neurologic recovery after dexamethasone initiation | 10 (29.41) | 7 (20.59) | 15 (44.12) | 2 (5.88) | 0 (0.00) | 34 |
| Discontinuation of immunotherapy because of dexamethasone dependence | 2 (5.88) | 1 (2.94) | 10 (29.41) | 17 (50.00) | 4 (11.76) | 34 |

**Supplementary tables**

| **Table S2** Table showing the preferred treatment of the different specialties for case 1, and the preferred treatment in the case of eight factors changing the case. | | | | | | | | | |
| --- | --- | --- | --- | --- | --- | --- | --- | --- | --- |
|  | Total number | Less edema | Larger tumor size | Intratumoral hemorrhage | Neurologic worsening (not responsive to DXM) | Presence of epileptic seizures | Radio- sensitive tumor | Neurologic recovery after DXM initiation | Discontinuation of immune-therapy because of DXM dependence |
| Radiotherapy | | | | | | | | | |
| Neurologist | 8 | RT: 3  N: 3  S: 1 * | RT: 0  N: 0  S: 8 | RT: 0  N: 6  S: 2 | RT: 1  N: 2  S: 5 | RT: 0  N: 7  S: 1 | RT: 5  N: 2  S: 1 | RT: 3  N: 5  S: 0 | RT: 0  N: 2  S: 6 |
| Oncologist | 4 | RT: 3  N: 1  S: 0 | RT: 0  N: 1  S: 3 | RT: 0  N: 2  S: 2 | RT: 0  N: 1  S: 3 | RT: 0  N: 2  S: 2 | RT: 4  N: 0  S: 0 | RT: 3  N: 1  S: 0 | RT: 1  N: 1  S: 2 |
| Neurosurgeon | 4 | RT: 3  N: 1  S: 0 | RT: 2  N: 1  S: 1 | RT: 1  N: 3  S: 0 | RT: 1  N: 2  S: 1 | RT: 1  N: 2  S: 1 | RT: 3  N: 1  S: 0 | RT: 3  N: 1  S: 0 | RT: 1  N: 1  S: 2 |
| Radiation oncologist | 9 | RT: 3  N: 5  S: 0 * | RT: 1  N: 1  S: 7 | RT: 2  N: 3  S: 3 * | RT: 2  N: 1  S: 6 | RT: 1  N: 7  S: 1 | RT: 6  N: 3  S: 0 | RT: 5  N: 4  S: 0 | RT: 1  N: 5  S: 3 |
| Surgery | | | | | | | | | |
| Neurologist | 2 | RT: 0  N: 2  S: 0 | RT: 0  N: 1  S: 1 | RT: 0  N: 1  S: 1 | RT: 0  N: 0  S: 2 | RT: 0  N: 1  S: 1 | RT: 1  N: 1  S: 0 | RT: 1  N: 1  S: 0 | RT: 0  N: 0  S: 2 |
| Oncologist | 1 | RT: 0  N: 1  S: 0 | RT: 0  N: 1  S: 0 | RT: 0  N: 0  S: 1 | RT: 0  N: 0  S: 1 | RT: 0  N: 1  S: 0 | RT: 1  N: 0  S: 0 | RT: 0  N: 1  S: 0 | RT: 0  N: 0  S: 1 |
| Neurosurgeon | 3 | RT: 2  N: 0  S: 1 | RT: 2  N: 0  S: 1 | RT: 0  N: 2  S: 1 | RT: 1  N: 0  S: 2 | RT: 0  N: 1  S: 2 | RT: 1  N: 1  S: 1 | RT: 0  N: 1  S: 2 | RT: 0  N: 0  S: 3 |
| Radiation oncologist | 1 | RT: 0  N: 1  S: 0 | RT: 0  N: 0  S: 1 | RT: 0  N: 1  S: 0 | RT: 1  N: 0  S: 0 | RT: 0  N: 1  S: 0 | RT: 0  N: 0  S: 1 | RT: 1  N: 0  S: 0 | RT: 0  N: 0  S: 1 |

One respondent mentioned that both treatment options were applicable (2.94%) and one respondent (2.94%) recommended to start with dexamethasone and then decide what the preferred treatment would be.

*DXM* dexamethasone, *RT* radiotherapy/radiosurgery, *N* neutral, *S* surgery

* The number of respondents doesn’t add up to the total number of respondents in the second column as not all respondents answered the question.

| **Table S3** Statements about factors influencing treatment decisions in case 2 | | | | | | |
| --- | --- | --- | --- | --- | --- | --- |
| Would your treatment change in case of: | Radiotherapy most preferred | Radiotherapy preferred | Neutral | Surgery preferred | Surgery most preferred | Total number of respondents |
| More edema | 3 (8.82) | 2 (5.88) | 15 (44.12) | 8 (23.53) | 6 (17.65) | 34 |
| Larger tumor size (> 4cm) | 1 (2.94) | 1 (2.94) | 2 (5.88) | 16 (47.06) | 14 (41.18) | 34 |
| Intratumoral hemorrhage | 3 (9.09) | 2 (6.06) | 15 (45.45) | 12 (36.36) | 1 (3.03) | 33 |
| Neurologic worsening (not responsive to dexamethasone) | 1 (2.94) | 3 (8.82) | 11 (32.35) | 12 (35.29) | 7 (20.59) | 34 |
| Presence of epileptic seizures | 2 (5.88) | 1 (2.94) | 24 (70.59) | 7 (20.59) | 0 (0.00) | 34 |
| Radiosensitive tumor | 5 (14.71) | 12 (35.29) | 15 (44.12) | 1 (2.94) | 1 (2.94) | 34 |
| Neurologic recovery after dexamethasone initiation | 2 (5.88) | 4 (11.76) | 17 (50.00) | 8 (23.53) | 3 (8.82) | 34 |
| Discontinuation of immunotherapy because of dexamethasone dependence | 1 (2.94) | 1 (2.94) | 12 (35.29) | 12 (35.29) | 8 (23.53) | 34 |

| **Table S4** Table showing the preferred treatment of the different specialties for case 2, and the preferred treatment in the case of eight factors changing the case. | | | | | | | | | |
| --- | --- | --- | --- | --- | --- | --- | --- | --- | --- |
|  | Total number | More edema | Larger tumor size | Intratumoral hemorrhage | Neurologic worsening (not responsive to DXM) | Presence of epileptic seizures | Radio- sensitive tumor | Neurologic recovery after DXM initiation | Discontinuation of immune-therapy because of DXM dependence |
| Radiotherapy | | | | | | | | | |
| Neurologist | 1 (9.09%) | R: 0  N: 0  S: 1 | R: 0  N: 0  S: 1 | R: 0  N: 0  S: 1 | R: 0  N: 1  S: 0 | R: 0  N: 1  S: 0 | R: 1  N: 0  S: 0 | R: 0  N: 1  S: 0 | R: 0  N: 0  S: 1 |
| Oncologist | 3 (60%) | R: 0  N: 2  S: 1 | R: 0  N: 1  S: 2 | R: 0  N: 0  S: 3 | R: 0  N: 1  S: 2 | R: 0  N: 2  S: 1 | R: 3  N: 0  S: 0 | R: 1  N: 1  S: 1 | R: 0  N: 1  S: 2 |
| Neurosurgeon | 3 (42.86%) | R: 2  N: 1  S: 0 | R: 2  N: 0  S: 1 | R: 3  N: 0  S: 0 | R: 1  N: 1  S: 1 | R: 2  N: 1  S: 0 | R: 3  N: 0  S: 0 | R: 2  N: 0  S: 1 | R: 1  N: 1  S: 1 |
| Radiation oncologist | 3 (27.27%) | R: 2  N: 1  S: 0 | R: 0  N: 1  S: 2 | R: 2  N: 1  S: 0 | R: 2  N: 1  S: 0 | R: 1  N: 2  S: 0 | R: 2  N: 1  S: 0 | R: 1  N: 2  S: 0 | R: 1  N: 2  S: 0 |
| Surgery | | | | | | | | | |
| Neurologist | 10 (90.91%) | R: 0  N: 6  S: 4 | R: 0  N: 0  S: 10 | R: 0  N: 7  S: 3 | R: 0  N: 2  S: 8 | R: 0  N: 8  S: 2 | R: 5  N: 4  S: 1 | R: 1  N: 5  S: 4 | R: 0  N: 2  S: 8 |
| Oncologist | 2 (40%) | R: 0  N: 0  S: 2 | R: 0  N: 0  S: 2 | R: 0  N: 1  S: 1 | R: 0  N: 0  S: 2 | R: 0  N: 2  S: 0 | R: 0  N: 2  S: 0 | R: 0  N: 2  S: 0 | R: 0  N: 1  S: 1 |
| Neurosurgeon | 4 (57.14%) | R: 0  N: 1  S: 3 | R: 0  N: 0  S: 4 | R: 0  N: 3  S: 0 * | R: 0  N: 3  S: 1 | R: 0  N: 2  S: 2 | R: 1  N: 2  S: 1 | R: 0  N: 1  S: 3 | R: 0  N: 1  S: 3 |
| Radiation oncologist | 8 (72.73%) | R: 1  N: 4  S: 3 | R: 0  N: 0  S: 8 | R: 0  N: 3  S: 5 | R: 1  N: 2  S: 5 | R: 0  N: 6  S: 2 | R: 2  N: 6  S: 0 | R: 1  N: 5  S: 2 | R: 0  N: 4  S: 4 |

*DXM* dexamethasone, *RT* radiotherapy/radiosurgery, *N* neutral, *S* surgery

* The number of respondents doesn’t add up to the total number of respondents in the second column as not all respondents answered the question.

| Table S5 Statements about factors influencing treatment decisions in case 3 | | | | | | |
| --- | --- | --- | --- | --- | --- | --- |
| Would your treatment change in case of: | Radiotherapy most preferred | Radiotherapy preferred | Neutral | Surgery preferred | Surgery most preferred | Total number of respondents |
| More edema | 3 (9.38) | 3 (9.38) | 16 (50.00) | 9 (28.13) | 1 (3.13) | 32 |
| Larger tumor size | 3 (9.09) | 1 (3.03) | 5 (15.15) | 17 (51.52) | 7 (21.21) | 33 |
| Intratumoral hemorrhage | 3 (9.38) | 2 (6.25) | 17 (53.13) | 9 (28.13) | 1 (3.13) | 32 |
| Neurologic worsening (not responsive to dexamethasone) | 2 (6.06) | 3 (9.09) | 7 (21.21) | 17 (51.52) | 4 (12.12) | 33 |
| Presence of epileptic seizures | 2 (6.06) | 1 (3.03) | 23 (69.70) | 7 (21.21) | 0 (0.00) | 33 |
| Radiosensitive tumor | 5 (15.15) | 14 (42.42) | 12 (36.36) | 1 (3.03) | 1 (3.03) | 33 |
| Neurologic recovery after dexamethasone initiation | 6 (18.18) | 10 (30.30) | 11 (33.33) | 4 (12.12) | 2 (6.06) | 33 |
| Discontinuation of immunotherapy because of dexamethasone dependence | 2 (6.06) | 1 (3.03) | 8 (24.24) | 15 (45.45) | 7 (21.21) | 33 |

| **Table S6** Table showing the preferred treatment of the different specialties for case 3, and the preferred treatment in the case of eight factors changing the case. | | | | | | | | | |
| --- | --- | --- | --- | --- | --- | --- | --- | --- | --- |
|  | Total number | More edema | Larger tumor size | Intratumoral hemorrhage | Neurologic worsening (not responsive to DXM) | Presence of epileptic seizures | Radio- sensitive tumor | Neurologic recovery after DXM initiation | Discontinuation of immune-therapy because of DXM dependence |
| Radiotherapy | | | | | | | | | |
| Neurologist | 6 | R: 0  N: 5  S: 1 | R: 0  N: 0  S: 6 | R: 0  N: 4  S: 2 | R: 1  N: 1  S: 4 | R: 0  N: 4  S: 2 | R: 4  N: 2  S: 0 | R: 2  N: 2  S: 2 | R: 0  N: 1  S: 5 |
| Oncologist | 4 | R: 0  N: 3  S: 1 | R: 0  N: 2  S: 2 | R: 0  N: 2  S: 2 | R: 0  N: 0  S: 4 | R: 0  N: 4  S: 0 | R: 3  N: 1  S: 0 | R: 3  N: 1  S: 0 | R: 0  N: 1  S: 3 |
| Neurosurgeon | 4 | R: 3  N: 1  S: 0 | R: 3  N: 0  S: 1 | R: 3  N: 1  S: 0 | R: 2  N: 1  S: 1 | R: 2  N: 2  S: 0 | R: 3  N: 1  S: 0 | R: 3  N: 1  S: 0 | R: 2  N: 0  S: 2 |
| Radiation oncologist | 7 | R: 2  N: 3  S: 2 | R: 1  N: 1  S: 5 | R: 1  N: 4  S: 2 | R: 2  N: 1  S: 4 | R: 1  N: 5  S: 1 | R: 4  N: 3  S: 0 | R: 4  N: 2  S: 1 | R: 1  N: 3  S: 3 |
| Surgery | | | | | | | | | |
| Neurologist | 5 | R: 0  N: 3  S: 2 | R: 0  N: 1  S: 4 | R: 0  N: 2  S: 2 * | R: 0  N: 1  S: 4 | R: 0  N: 5  S: 0 | R: 2  N: 2  S: 1 | R: 1  N: 3  S: 1 | R: 0  N: 1  S: 4 |
| Oncologist | 1 | R: 0  N: 0  S: 1 | R: 0  N: 0  S: 1 | R: 0  N: 0  S: 1 | R: 0  N: 0  S: 1 | R: 0  N: 0  S: 1 | R: 1  N: 0  S: 0 | R: 1  N: 0  S: 0 | R: 0  N: 0  S: 1 |
| Neurosurgeon | 3 | R: 0  N: 0  S: 2 * | R: 0  N: 1  S: 2 | R: 0  N: 3  S: 0 | R: 0  N: 1  S: 2 | R: 0  N: 1  S: 2 | R: 1  N: 1  S: 1 | R: 1  N: 0  S: 2 | R: 0  N: 0  S: 3 |
| Radiation oncologist | 2 | R: 1  N: 1  S: 0 | R: 1  N: 1  S: 0 | R: 1  N: 1  S: 0 | R: 0  N: 2  S: 0 | R: 0  N: 2  S: 0 | R: 1  N: 1  S: 0 | R: 0  N: 2  S: 0 | R: 0  N: 2  S: 0 |

*DXM* dexamethasone, *RT* radiotherapy/radiosurgery, *N* neutral, *S* surgery

* The number of respondents doesn’t add up to the total number of respondents in the second column as not all respondents answered the question.
